# Emulating a randomized clinical trial with real-world data to evaluate the effect of antidepressant use in PTSD patients with high suicide risk

**DOI:** 10.3389/fpsyt.2024.1526488

**Published:** 2025-01-28

**Authors:** Oshin Miranda, Xiguang Qi, M. Daniel Brannock, Ryan Whitworth, Thomas Kosten, Neal David Ryan, Gretchen L. Haas, Levent Kirisci, LiRong Wang

**Affiliations:** ^1^ Department of Pharmaceutical Sciences/School of Pharmacy, University of Pittsburgh, Pittsburgh, PA, United States; ^2^ RTI International, Durham, NC, United States; ^3^ Menninger Department of Psychiatry, Baylor College of Medicine, Houston, TX, United States; ^4^ Department of Psychiatry, School of Medicine, University of Pittsburgh, Pittsburgh, PA, United States; ^5^ Department of Psychology, Dietrich School of Arts and Sciences, University of Pittsburgh, Pittsburgh, PA, United States; ^6^ VISN 4 Mental Illness Research, Education and Clinical Center (MIRECC), VA Pittsburgh Healthcare System, Pittsburgh, PA, United States

**Keywords:** social determinants of health, post-tramatic stress disorder, suicide-related behavior, antidepressant, clinical trial emulation

## Abstract

**Introduction:**

Post-Traumatic Stress Disorder (PTSD) entails behavioral changes with increased risk of suicide, and there is no consensus on the preferred antidepressants for treatment of those PTSD patients who are at elevated risk for suicide.

**Methods:**

We conducted a clinical trial emulation study comparing suicide-related events (SREs) among those patients’ initiating antidepressants within 60 days after a qualifying SRE. Patients were followed from initiation of antidepressant until any of the following: treatment cessation, switching, death, or loss to follow-up. The outcome is a new onset of an SRE.

**Results:**

Citalopram exhibited a significantly fewer case with new SREs compared to other most used antidepressants such as venlafaxine, duloxetine, and mirtazapine–even after adjusting for multiple comparisons and other covariants.

**Discussion:**

Findings suggest potential risks associated with certain antidepressants in the PTSD population, emphasizing cautious prescription considerations.

## 1 Introduction

Exposure to traumatic events is common to various psychiatric disorders, including major depression, bipolar disorder, psychosis, anxiety, personality disorders, and trauma-related conditions such as posttraumatic stress disorder (PTSD) (1). PTSD is a particularly severe psychiatric condition that, by definition, emerges following the experience of a life-threatening or deeply traumatic event. Symptoms of PTSD include intrusive memories, hypervigilance, feelings of guilt or shame, psychological distress, disturbed sleep, avoidance of trauma reminders, and negative changes in thinking, mood, and cognition (2). These symptoms may appear soon after the traumatic event or be delayed, with onset and duration varying greatly among individuals. Globally, it is estimated that 70% of adults have encountered a traumatic event, although among these, only 6% develop PTSD (3). The prevalence is notably higher in groups exposed to severe trauma, such as military veterans, with about 25% potentially suffering from the disorder (4). The profound impact of PTSD on the everyday functioning of individuals highlights the necessity for accurate diagnosis, effective treatment, and ongoing support.

The Food and Drug Administration (FDA) has approved certain antidepressants, particularly paroxetine and sertraline, as primary pharmacological treatments for PTSD (5). Additional medications, including prazosin (an α1-blocker) and antipsychotics like quetiapine and risperidone, have shown significant efficacy for specific PTSD symptoms, compared to placebo (6). However, some treatments have not effectively reduced the increased risk of suicide in these patients (7).

PTSD markedly raises the risk of both suicide attempts and suicide deaths (8, 9). This disorder encompasses various forms of suicidality, such as suicidal thoughts and attempts (10). Suicidal behavior is prevalent among individuals with PTSD, as supported by a meta-analysis of prospective studies (11, 12). While PTSD has been associated with suicide-related events (SREs, (defined to include suicidal thoughts, attempts, and death by suicide), the precise nature and extent of this relationship are not yet fully understood (8, 9), pointing to the urgent need for effective intervention strategies in PTSD treatment (8).

Timely and effective intervention is crucial to alleviating distress, preventing chronic conditions, and reducing the burden on healthcare systems. Current evidence-based guidelines recommend psychological therapies such as Cognitive Processing Therapy (CPT), a trauma-focused cognitive-behavioral therapy, Prolonged Exposure (PE) therapy and eye movement desensitization and reprocessing (EMDR), along with pharmacological treatments like selective serotonin reuptake inhibitors (SSRIs) for managing PTSD (13). These guidelines prioritize psychotherapy over medication, reserving antipsychotic drugs for severe or treatment-resistant cases (14). While the efficacy of psychiatric admission in preventing suicide is still uncertain, individuals with PTSD who exhibit suicidality or suicide attempts may require hospitalization or crisis intervention (15). Evidence supporting the use of anti-anxiety and antidepressant medications to reduce the risk of repeated self-harm is limited. However, there is moderately strong evidence for the effectiveness of psychosocial interventions, such as cognitive behavioral therapy (CBT), in the general population of individuals who self-harm, although not specifically for those with PTSD. Given that suicidality is a significant predictor of suicide, it is essential to examine the characteristics and treatment pathways of PTSD patients with suicidal tendencies (16). Our recent 2020 study on SREs in PTSD patients with bipolar disorder found that the use of some antidepressants like Trazodone is a significant predictor of increased SREs while Sertraline use is associated with reduced SREs (17). Antidepressants are commonly prescribed for major depressive disorder, anxiety disorders, substance use disorders, and other chronic pain conditions (18).

Social determinants significantly influence health outcomes, shaping biomedical results and affecting healthcare utilization. Understanding the impact of factors such as relationship breakdowns, financial instability, legal problems, and childhood adversity is critical, as these elements are intricately linked to suicidal behaviors (19–24). Therefore, integrating social and behavioral data into electronic medical records (EMRs) is crucial. In the United States, incorporating social determinants of health (SDoH) into EMRs has been a gradual process. Despite foundational research emphasizing the importance of SDoH in population health for decades, the National Academy of Medicine only recommended collecting these determinants in 2014 (25). This delay may be due to the historical focus of healthcare systems on individual biological mechanisms and health behaviors, often overlooking broader social forces. To improve patient outcomes and reduce healthcare costs, it is vital to examine SDoH and their associations with outcomes like suicide ideation, attempts, and death.

Antidepressants are considered promising for PTSD treatment. Research has shown that paroxetine (SSRI), sertraline (SSRI), and venlafaxine (SNRI) are particularly effective compared to other antidepressants (5). However, these medications often cause side effects before therapeutic benefits are felt, leading to antidepressant discontinuation syndrome or withdrawal syndrome (5). In 2004, the US FDA issued a black box warning regarding the use of antidepressants in children and adolescents, citing potential risks of suicidal ideation and attempts. This warning was extended in 2006 to include young adults aged 18 to 25 years. These actions were based on a meta-analysis of adverse event reports (AERs) from 25 clinical randomized controlled trials conducted by pharmaceutical companies during the approval process for newer classes of antidepressants, specifically SSRIs and serotonin-norepinephrine reuptake inhibitors (SNRIs). The analysis revealed a significant overall odds ratio (OR) of 1.78 (95% CI, 1.14–2.77), indicating a higher rate of suicidal thoughts and behaviors in children receiving active antidepressant treatment compared to those receiving placebo. The extension of the warning to young adults was supported by a second meta-analysis of AERs from 372 RCTs involving 99,839 adult patients. This analysis showed a trend toward significance in young adults aged 18 to 24 years (OR=1.62; 95% CI, 0.97–2.71), while there was a significantly decreased risk in adults aged 25 to 64 years (OR=0.79; 95% CI, 0.64–0.98) and geriatric patients aged 65 years and older (OR=0.37; 95% CI, 0.18–0.76) (26). However, a subsequent FDA analysis focused on prospectively measured suicidal thoughts and behaviors in youth studies found no evidence of an increased risk associated with antidepressant treatment (27). This analysis centered on study endpoints and did not utilize complete longitudinal data available in these studies, with no parallel analysis presented for adult studies. Currently, there is no consensus on the preferred antidepressants for treating PTSD patients with high SRE risk. This observational study aims to identify specific antidepressants associated with a reduced incidence of SREs.

Randomized clinical trials (RCTs) are the gold standard for assessing medication efficacy. However, RCTs face limitations in evaluating all aspects of a drug’s effects across diverse populations. Therefore, decision-makers look to real-world evidence (RWE) to understand how medical products perform in real-world clinical settings. RWE is derived from non-randomized data, including electronic medical records. Despite the potential of RWE to inform clinical practice, the variable methodological rigor of RWE studies poses challenges in drawing actionable insights and causal conclusions. To validate RWE studies, their results need to be compared against well-conducted RCTs, the established standard for causal inference. Previous comparisons between published RCTs and non-randomized RWE studies have yielded mixed results. However, these comparisons often involved RWE studies that were not designed to emulate RCTs, introducing variability and complicating result assessment (28).

To establish robust clinical guidelines, comprehensive evidence on potential effects and relevant outcomes is essential. While RCTs are the gold standard, practical constraints like time, cost, and ethical considerations often limit their feasibility. As an economical alternative, observational data is frequently used, despite being prone to selection bias and immortal-time bias, which RCTs mitigate (29–31). This study employs emulation methods to address these biases in observational data. Clinical trial emulation frameworks were first proposed by Hernan and Robin in 2016 (32) and was further updated by Hernan in 2022 (33). And since then, this method has been widely adopted in observational studies with more than 2000 citations as of today. Emulating RCTs involves specifying a target trial and developing a protocol to eliminate selection bias, facilitating comparisons between individuals initiating different medications. To minimize immortal-time bias, the analysis used in this study ensures consistent timing for eligibility and treatment initiation, similar to RCTs (34). This study aims to reduce potential biases by using emulation methods, leveraging EMR data to examine SDoH profiles of PTSD patients, and addressing gaps in understanding the relationship between antidepressant treatments and emergence of suicide risk events. This study also aims to explore the relationship between suicidality and antidepressant treatments in PTSD patients to reduce the risk of SREs. Specifically, the study explores two critical aspects: the emergence of SREs associated with antidepressant use and the relationship between antidepressant treatments and SRE risk within six months following the prior suicide event that qualified for study inclusion. This approach allows for a deeper examination of treatment related risks, contributing a unique insight to the existing literature on PTSD management and suicide prevention.

## 2 Materials and methods

### 2.1 Description of data source

We analyzed data from January 2004 to October 2020 using the Neptune system at the University of Pittsburgh, which manages patient electronic medical records from the UPMC health system for research purposes (rio.pitt.edu/services). The database includes comprehensive demographic information, diagnoses, encounters, medication prescriptions, prescription fill history, and laboratory tests. The study received approval from the Institutional Review Board (IRB) at the University of Pittsburgh (STUDY19020153, approved on March 13, 2019). The IRB concluded that the research activity does not meet the definition of human subject research as outlined by the U.S. Department of Health and Human Services (DHHS) and Food and FDA regulations.

### 2.2 Methodological approach to inclusion/exclusion and endpoints/follow-up

Baseline eligibility criteria included initiating an antidepressant after the diagnosis of PTSD (qualifying event) and within six months following a recent SRE (qualifying event). Participants must have had no prior use of antidepressants in the 60 days preceding the study (washout period) and at least one year of recorded history before the initiation of antidepressant of interest in the electronic medical records. The beginning of the one-year record can be before or after the PTSD diagnosis, but the end of the one-year record should be the enrollment date. This date is after the PTSD diagnosis. If these criteria were met, patients were followed until the onset of the first suicide-related event (primary outcome) or until they were lost to follow-up, which included stopping the use of the antidepressant, switching to another antidepressant, patient data no longer being accessible, or reaching the study’s end time.

### 2.3 Incorporation of social determinants of health

For each PTSD patient, we integrated individual-level and neighborhood-level social determinants of health data into our analysis. Individual-level features, such as race, age, and gender, were extracted from the electronic medical records and demographic information. Neighborhood-level features included racial segregation, socio-economic status, percentage of non-citizens, person of color index, normalized difference vegetation index, aridity index, percentage of male widowers, percentage of U.S. citizens, households with limited English proficiency, income segregation, percentage of same-sex marriages, urban index, percentage of separated individuals, and percentage of households with transportation barriers. These features were separately calculated using their respective formulas and extracted from the American Community Survey (ACS), as done in our previous studies (35, 36).

### 2.4 Emulation of target trials

To be considered for inclusion in this trial, eligible PTSD patients met the following inclusion criteria: a chart diagnosis of PTSD, a chart-record of a recent SRE (within six months), and no antidepressant in the 60 days preceding enrollment. Upon enrollment, patients were randomly allocated to one of the compared antidepressant arms within the target trials. The selected antidepressants for our investigation include bupropion, citalopram, duloxetine, fluoxetine, mirtazapine, sertraline, trazodone, and venlafaxine. Patients retained for the analysis could have been prescribed other medications for addressing concurrent health conditions. For each patient, the course of the trial for a patient was defined as concluded at the earliest point among the following: a) a patient has no SRE at the end of the study period (i.e., administrative censoring), b) failure to return for a study visit (i.e., lost to follow-up), c) stops using the antidepressant of interest (i.e. no records of use within two months of initial prescription), d) switches to another antidepressant, or e) experiences a new onset of SRE. The primary outcome under investigation is the onset of SREs.

To address potential biases inherent in observational data, this study emulated randomized controlled trials similar to the work conducted by Danaei and colleagues to mitigate selection bias and immortal-time bias (37). Specifically, the analysis aligned eligibility criteria and treatment initiation timing to ensure consistent observation periods, mimicking the design of randomized controlled trials. These methods were implemented to strengthen causal inference and provide robust comparisons between individuals initiating different antidepressant medications. Throughout the study duration, it was imperative to assess confounding variables, which are detailed in the Table of Methods section and categorized based on ICD-9 and ICD-10 codes (refer to **Appendix A**). Additionally, adhering to the eligibility criteria, participants required a minimum of one year of continuous recording in the UPMC medical records and at least one medical visit within a year of the trial’s initiation. Monthly trials were extracted from the UPMC EMR database, spanning January 2004 to October 2020, covering a total of 197 months. Patients could be enrolled in the study multiple times, provided they observed a 60-day washout period for antidepressant use and met all the inclusion criteria. Eligible patients were assigned to specific target trial arms based on the antidepressant utilized. The efficacy of these target trials for antidepressants was subsequently compared, with the primary outcome focusing on experienced SREs. The study duration ceased if the medication of interest was discontinued or if the patient’s EMR data became unavailable (due to loss to follow-up or death). In simpler terms, patients were right-censored if they had no SREs at the study period’s conclusion (administrative censoring), if they failed to return for a study visit (lost to follow-up), if they ceased using the antidepressant of interest (with no records of use within three months), or if they transitioned to another antidepressant.

### 2.5 Implementation of per-protocol analysis

We applied a per-protocol analysis to ensure that all participants strictly adhered to the prescribed treatment regimen. The comparative study evaluated the impact of two drugs on the outcomes of cohorts that completed their initially assigned treatments. It is important to acknowledge that this analysis might introduce biases due to baseline confounders and post-baseline, time-varying confounders. Following the methodology of Danaei and colleagues, we employed a pooled logistic regression model to estimate the treatment effect, using inverse probability weighting to create a population where treatment independence from prognostic factors is preserved (34, 37). Baseline information, which includes 12 categories of mental disorders (see **Appendix B**), age, gender, and the number of emergency department visits within one year before enrollment, is presented in **Table 1** (38). To evaluate the impact of social determinants of health and concomitant medications, an additional analysis was performed. This analysis adjusted for (1) both individual and neighborhood-level social determinants of health and (2) the most frequently used drugs of the central nervous system: benzodiazepines, antipsychotics and pain medications. In our emulation study, we employed a pooled logistic regression model to account for censoring effects. We did not consider the transition between paired antidepressants, as this scenario was appropriately represented by the censored model. Robust variances were used to calculate conservative 95% confidence intervals, and inverse probability weights were truncated to their 99th percentile. These options were implemented using code available at www.hsph.harvard.edu/causal/software. Additionally, the Firth method in logistic regression was applied to address rare events or complete separation. Datasets were prepared using Python (39), and the final analyses were conducted using SAS 9.4 (SAS Institute Inc., Cary, NC, USA) (40). To reduce the risk of Type I error inflation from multiple hypothesis tests, the false discovery rate (FDR) q-value was controlled at 0.05. FDR calculations were performed using the “p.adjust” function in the base package of R version 4.0.2 (41).

**Table 1:** Baseline characteristics of the patients included in this study are categorized as follows: Level 0 indicates absence of a diagnosis fitting under the category, while Level 1 indicates presence of such a diagnosis.

	Bupropion	Citalopram	Duloxetine	Fluoxetine	Mirtazapine	Sertraline	Trazodone	Venlafaxine	p value
**n**	115	152	97	176	125	256	385	115	
**Age_b (mean (SD))**	34.66 (13.58)	38.35 (13.40)	40.41 (14.45)	32.36 (13.45)	40.18 (12.59)	34.81 (14.91)	37.38 (12.30)	37.49 (13.37)	<0.001
**Gender = Male (%)**	50 (43.5)	62 (40.8)	27 (27.8)	67 (38.1)	65 (52.0)	101 (39.5)	171 (44.4)	38 (33.0)	0.007
**ED_Vistis_3Month_b (mean (SD))**	1.09 (1.88)	1.31 (2.26)	1.69 (2.65)	1.03 (2.69)	1.26 (1.68)	1.01 (2.04)	1.31 (2.13)	1.07 (2.25)	0.194
**Category 1 (Alcohol and drug-related disorders) = 1 (%)**	35 (30.4)	50 (32.9)	28 (28.9)	44 (25.0)	50 (40.0)	71 (27.7)	144 (37.4)	36 (31.3)	0.03
**Category 2 (Schizophrenia and schizoid personality disorders) = 1 (%)**	12 (10.4)	10 (6.6)	11 (11.3)	16 (9.1)	10 (8.0)	31 (12.1)	64 (16.6)	12 (10.4)	0.024
**Category 3 (Mood disorders) = 1 (%)**	71 (61.7)	90 (59.2)	71 (73.2)	103 (58.5)	77 (61.6)	153 (59.8)	235 (61.0)	74 (64.3)	0.393
**Category 4 (Delusional and nonorganic psychoses) = 1 (%)**	8 (7.0)	3 (2.0)	1 (1.0)	6 (3.4)	8 (6.4)	16 (6.2)	25 (6.5)	4 (3.5)	0.109
**Category 5 (Anxiety and stress-related disorders) = 1 (%)**	41 (35.7)	64 (42.1)	51 (52.6)	61 (34.7)	46 (36.8)	102 (39.8)	168 (43.6)	50 (43.5)	0.095
**Category 6 (Personality disorders (excluding affective and schizoid types)) = 1 (%)**	18 (15.7)	19 (12.5)	24 (24.7)	23 (13.1)	10 (8.0)	25 (9.8)	57 (14.8)	17 (14.8)	0.012
**Category 7 (Sexual and gender identity disorders) = 1 (%)**	0 (0.0)	0 (0.0)	0 (0.0)	1 (0.6)	1 (0.8)	0 (0.0)	0 (0.0)	1 (0.9)	0.368
**Category 8 (Physiological and psychological factors affecting physical conditions) = 1 (%)**	1 (0.9)	0 (0.0)	0 (0.0)	2 (1.1)	2 (1.6)	0 (0.0)	0 (0.0)	0 (0.0)	0.07
**Category 9 (Special symptoms or syndromes not elsewhere classified) = 1 (%)**	3 (2.6)	5 (3.3)	3 (3.1)	8 (4.5)	1 (0.8)	7 (2.7)	5 (1.3)	10 (8.7)	0.004
**Category 10 (Dementias and mental disorders due to external conditions) = 1 (%)**	1 (0.9)	2 (1.3)	1 (1.0)	2 (1.1)	2 (1.6)	4 (1.6)	5 (1.3)	2 (1.7)	0.999
**Category 11 (Disorders specific to childhood and developmental issues) = 1 (%)**	14 (12.2)	12 (7.9)	12 (12.4)	24 (13.6)	6 (4.8)	33 (12.9)	48 (12.5)	10 (8.7)	0.177
**Category 12 (Intellectual disabilities) = 1 (%)**	3 (2.6)	2 (1.3)	3 (3.1)	1 (0.6)	1 (0.8)	11 (4.3)	12 (3.1)	3 (2.6)	0.248
**BZO_b (mean (SD))**	0.66 (0.86)	1.03 (1.09)	1.02 (0.98)	0.81 (1.02)	1.07 (1.07)	0.81 (0.89)	0.99 (0.97)	0.91 (1.03)	0.004
**antipsychotics_b (mean (SD))**	0.89 (0.90)	0.84 (1.01)	0.81 (0.79)	0.72 (0.91)	1.00 (1.01)	0.82 (0.98)	1.17 (1.19)	0.93 (0.87)	<0.001
**pain_med_b (mean (SD))**	0.78 (1.15)	0.96 (1.39)	1.72 (1.71)	0.81 (1.16)	1.06 (1.23)	0.86 (1.35)	0.90 (1.32)	0.86 (1.14)	<0.001

*Category 1: Alcohol and drug-related disorders, covering ICD-9 codes 291* (alcohol-induced mental disorders), 292* (drug-induced mental disorders), 303* (alcohol dependence syndrome), 304* (drug dependence), and 305* (nondependent abuse of drugs, excluding 305.1 for tobacco use disorder). Category 2: Schizophrenia and schizoid personality disorders, with ICD-9 codes 295* (schizophrenic disorders) and 301.2 (schizoid personality disorder). Category 3: Mood disorders, including ICD-9 codes 296* (episodic mood disorders), 298.0 (depressive type psychosis), 300.4 (dysthymic disorder), 301.1 (affective personality disorder), 309* (adjustment reaction), and 311* (depressive disorder NEC). Category 4: Delusional and nonorganic psychoses, containing ICD-9 codes 297* (delusional disorders) and 298* (other nonorganic psychoses, excluding 298.0). Category 5: Anxiety and stress-related disorders, represented by ICD-9 codes 308* (acute reaction to stress) and 300* (anxiety, dissociative, and somatoform disorders, excluding 300.4). Category 6: Personality disorders (excluding affective and schizoid types), covered by ICD-9 code 301* (personality disorders, excluding 301.1 and 301.2). Category 7: Sexual and gender identity disorders, under ICD-9 code 302*. Category 8: Physiological and psychological factors affecting physical conditions, with ICD-9 codes 306* (physiological malfunction due to mental factors) and 316* (psychic factors with other diseases). Category 9: Special symptoms or syndromes not elsewhere classified, represented by ICD-9 code 307*. Category 10: Dementias and mental disorders due to external conditions, including ICD-9 codes 290* (dementias), 293* (transient mental disorders due to external conditions), 294* (persistent mental disorders due to external conditions), and 310* (nonpsychotic mental disorders due to brain damage). Category 11: Disorders specific to childhood and developmental issues, with ICD-9 codes 299* (autistic disorder), 312* (conduct disturbance), 313* (emotional disturbances in childhood/adolescence), 314* (hyperkinetic syndrome of childhood), and 315* (developmental delays). Category 12: Intellectual disabilities, covering ICD-9 codes 317* (mild intellectual disabilities), 318* (other specified intellectual disabilities), and 319* (unspecified intellectual disabilities). Additionally, **Table A1** in **Appendix B** lists the specific drugs considered within the benzodiazepines, antipsychotics, and pain medication categories.

## 3 Results

**Figure 1:**
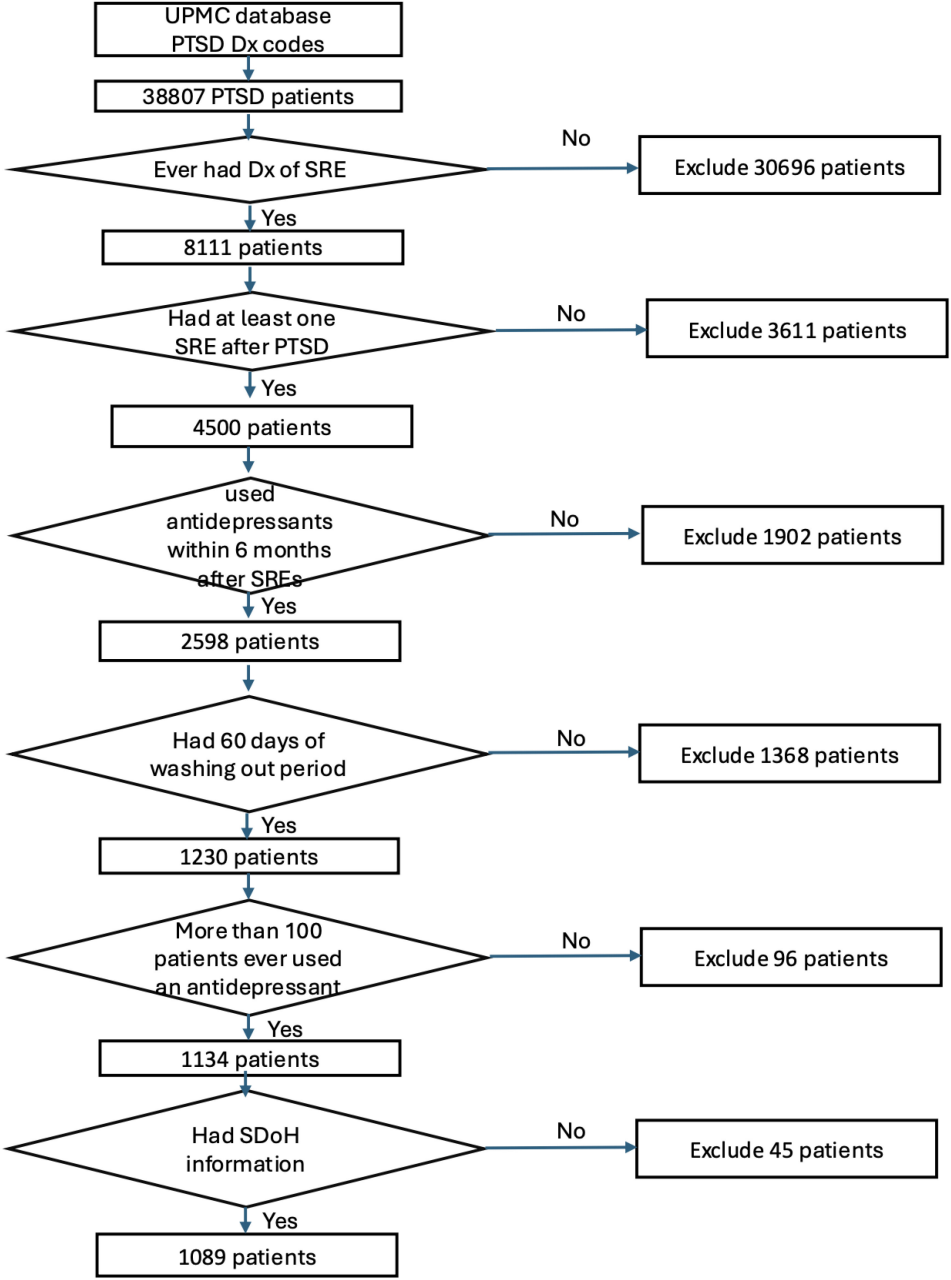
Participant screening and enrollment flowchart.

**Figure 2:**
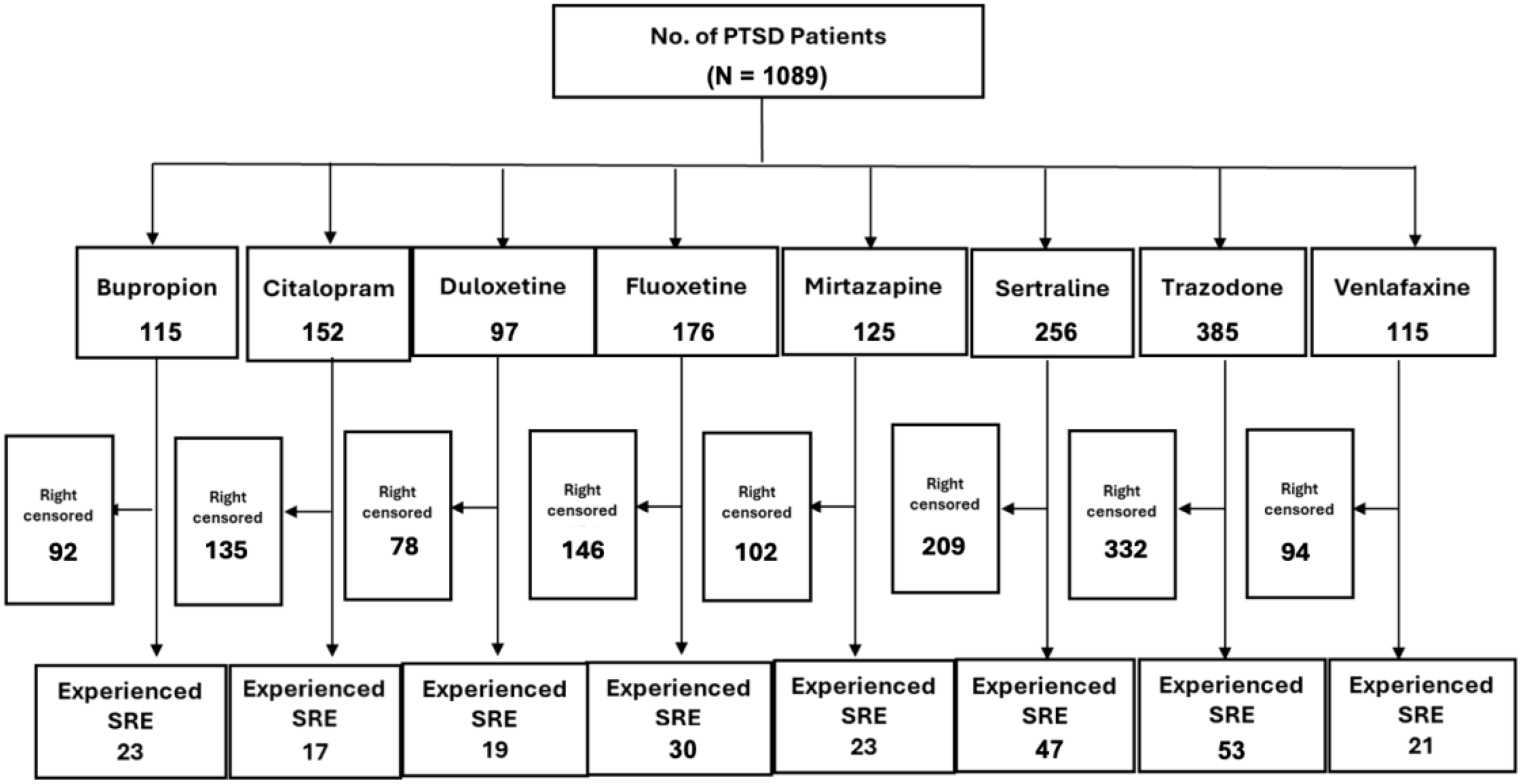
Emulation selection process overview.

**Figure 3:**
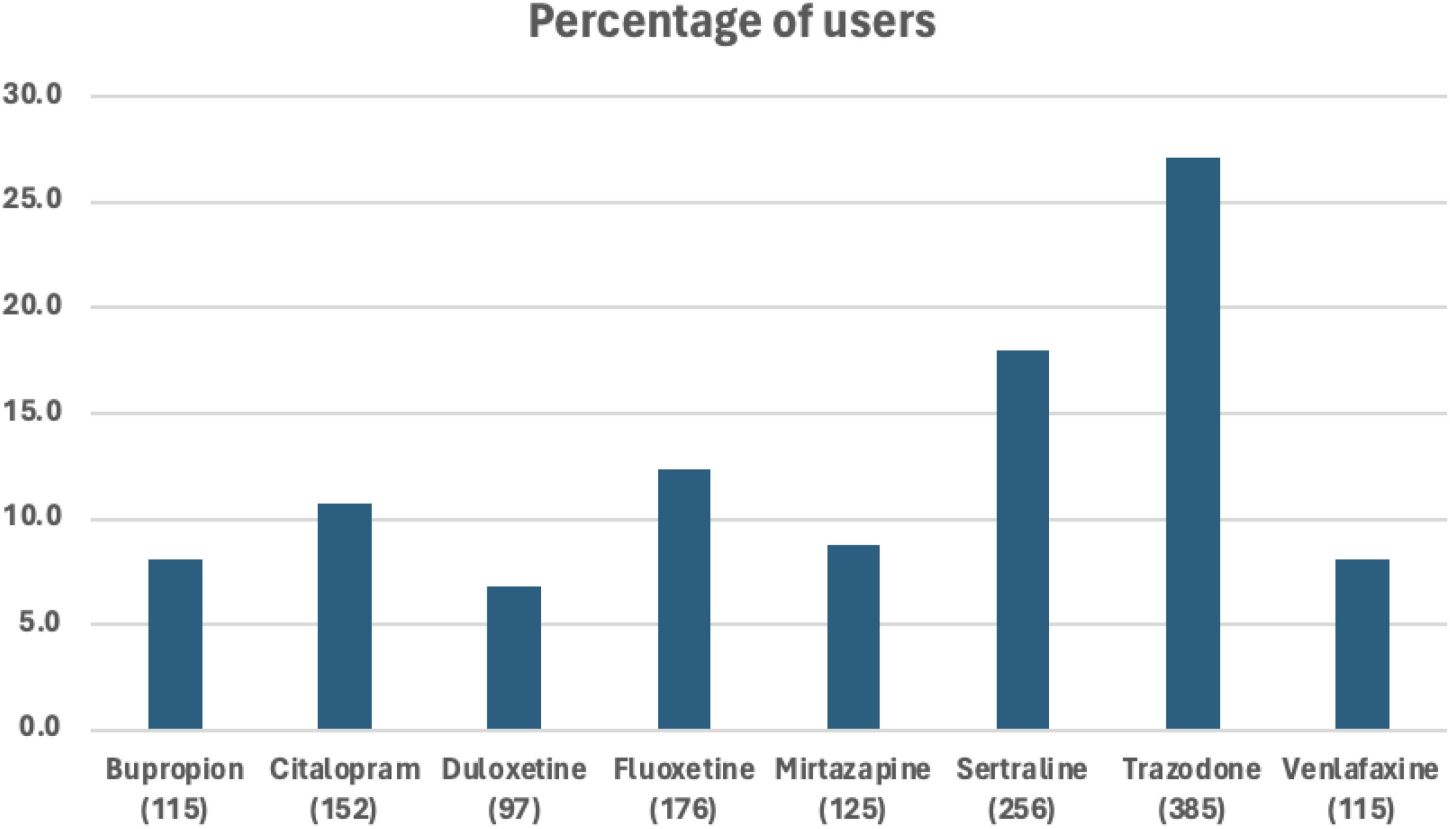
Percent of eligible patients with PTSD using a certain type of antidepressant.

Out of the 38,807 patients diagnosed with PTSD, our study included 1,089 patients who began taking antidepressants after their PTSD diagnosis (**Figure 1**). These patients need to have a SRE and complete washout period, with at least one year of documented history in electronic medical records. They were categorized into treatment groups and monitored until any of the following occurred: treatment discontinuation, switch to another drug within the same class, SRE, death, or loss to follow-up. **Figure 2** illustrates the emulation process. Trazodone emerged as the most frequently prescribed antidepressant, prescribed for 27.1% of the study cohort, while Duloxetine was the least prescribed, representing 6.8% of the sample (see **Figure 3**). Detailed baseline characteristics can be found in **Table 1**; **Table 2** outlines the distribution of antidepressant prescription among PTSD patients in our study.

**Table 2:** Number of patients, events, and unique patients, ratio and average number of months of follow-up for each antidepressant treatment respectively.

Drug name	No. of patients	No. of events	No. of unique patients	Ratio	Average months of follow-up(months)
Bupropion	115	23	100	0.23	4.70
Citalopram	152	17	136	0.12	4.59
Duloxetine	97	19	84	0.23	4.30
Fluoxetine	176	30	162	0.19	4.71
Mirtazapine	125	23	108	0.21	3.86
Sertraline	256	47	228	0.21	4.57
Trazodone	385	53	324	0.16	3.59
Venlafaxine	115	21	104	0.20	4.44

Out of the 1,089 eligible patients in our study, the average follow-up duration was 3.38 months, during which 233 events occurred. Among those 233 SRE events, 164 were suicidal ideations, 69 were suicide attempts and none were suicidal death. We adjusted outcomes for baseline characteristics, social determinants of health, and concurrent antidepressant therapy. The overall proportion of significant adverse events (SREs) was 16% (242 out of 1,295). The observed event rates for each antidepressant were as follows: bupropion 19% (23/115), citalopram 11% (17/152), duloxetine 19% (19/97), fluoxetine 17% (30/176), mirtazapine 18% (23/125), sertraline 20% (53/256), trazodone 14% (54/385), and venlafaxine 18% (21/115).

As detailed in **Table 3**, pairwise comparisons were conducted to assess the relative efficacy of each antidepressant in managing SREs. Notably, patients treated with citalopram experienced significantly fewer SREs compared to venlafaxine (p < 0.001), duloxetine (p = 0.001), and mirtazapine (p = 0.0008). Similarly, citalopram showed significant differences in SRE occurrence compared to venlafaxine, duloxetine, and mirtazapine, with adjusted p-values of < 0.0028, 0.0131, and 0.0112, respectively. These findings highlight the robust clinical significance of managing SREs with citalopram, even after adjusting for comorbidities, social determinants of health, and concurrent medications such as benzodiazepines, pain relievers, and antipsychotics.

**Table 3:** Head-to-head comparisons of antidepressants, adjusted for social determinants of health and concurrent PTSD medications, using truncating weights.

	Comorbidities				Comorbidities+SDOH				Comorbidities+SDOH+other Meds			
	OR [lb, up]	z	p-value	Adjusted FDR value	OR [lb, up]	z	p-value	Adjusted FDR value	OR [lb, up]	z	p-value	Adjusted FDR value
Fluoxetine vs. Sertraline	0.930 [1.655, 1.430]	-0.284	0.7765	0.9059	0.875 [1.636, 1.254]	-0.53	0.5971	0.7599	0.880 [1.623, 1.255]	-0.52	0.6028	0.8334
Fluoxetine vs. Venlafaxine	0.864 [2.059, 1.536]	-0.397	0.6912	0.9059	0.893 [1.904, 1.519]	-0.34	0.7309	0.8485	0.960 [1.954, 1.799]	-0.121	0.9036	0.9134
Fluoxetine vs. Bupropion	1.484 [1.978, 4.358]	1.135	0.2563	0.6524	1.351 [2.026, 3.699]	0.84	0.4038	0.6281	1.151 [1.978, 2.622]	0.406	0.6846	0.8334
Fluoxetine vs. Citalopram	1.707 [2.042, 5.954]	1.469	0.1419	0.6524	2.155 [1.883, 8.750]	2.38	0.0174	0.1218	2.234 [1.866, 9.318]	2.524	0.0116	0.065
Fluoxetine vs. Duloxetine	1.145 [2.065, 2.702]	0.364	0.7156	0.9059	1.401 [2.151, 4.221]	0.86	0.3884	0.6281	1.468 [2.316, 4.993]	0.896	0.3704	0.6482
Fluoxetine vs. Mirtazapine	0.969 [2.002, 1.876]	-0.092	0.927	0.9613	0.921 [1.950, 1.657]	-0.24	0.8103	0.8485	0.964 [1.960, 1.818]	-0.109	0.9134	0.9134
Fluoxetine vs. Trazodone	1.239 [1.699, 2.604]	0.791	0.4288	0.7504	1.077 [1.640, 1.902]	0.29	0.7689	0.8485	1.130 [1.634, 2.088]	0.487	0.6262	0.8334
Sertraline vs. Venlafaxine	1.042 [1.829, 1.988]	0.134	0.8933	0.9613	1.064 [1.883, 2.132]	0.19	0.8485	0.8485	1.068 [1.889, 2.155]	0.203	0.8389	0.9134
Sertraline vs. Bupropion	1.626 [1.766, 4.674]	1.673	0.0943	0.6524	1.784 [1.828, 5.818]	1.88	0.0597	0.2388	1.822 [1.826, 6.062]	1.954	0.0507	0.1775
Sertraline vs. Citalopram	2.232 [1.970, 9.826]	2.32	0.0203	0.1895	2.411 [1.881, 10.924]	2.73	0.0063	0.0588	2.255 [1.822, 9.272]	2.655	0.0079	0.0553
Sertraline vs. Duloxetine	1.564 [1.872, 4.577]	1.396	0.1627	0.6524	1.122 [1.964, 2.472]	0.34	0.7377	0.8485	1.051 [1.931, 2.132]	0.148	0.8826	0.9134
Sertraline vs. Mirtazapine	0.922 [1.679, 1.428]	-0.306	0.7599	0.9059	1.062 [1.820, 2.050]	0.2	0.845	0.8485	1.069 [1.782, 2.040]	0.228	0.8198	0.9134
Sertraline vs. Trazodone	1.166 [1.551, 2.109]	0.686	0.4925	0.8112	1.292 [1.527, 2.547]	1.19	0.2352	0.5987	1.379 [1.513, 2.872]	1.519	0.1288	0.3279
Venlafaxine vs. Bupropion	1.550 [1.988, 4.773]	1.249	0.2116	0.6524	1.390 [2.000, 3.865]	0.93	0.3517	0.62	1.467 [1.998, 4.293]	1.084	0.2783	0.5994
Venlafaxine vs. Citalopram	2.447 [1.921, 11.508]	2.687	0.0072	0.1895	3.435 [1.893, 22.332]	3.79	0.0001	0.0028	4.764 [2.016, 45.741]	4.363	<.0001	<.0028
Venlafaxine vs. Duloxetine	1.399 [1.948, 3.815]	0.988	0.323	0.6957	1.665 [1.960, 5.430]	1.49	0.1376	0.3853	1.946 [2.048, 7.752]	1.821	0.0686	0.2134
Venlafaxine vs. Mirtazapine	1.194 [1.916, 2.729]	0.534	0.5934	0.8429	1.293 [2.065, 3.449]	0.7	0.4872	0.6987	1.225 [1.970, 2.954]	0.586	0.5577	0.8334
Venlafaxine vs. Trazodone	1.288 [1.766, 2.933]	0.872	0.3834	0.7504	1.342 [1.697, 3.056]	1.09	0.276	0.62	1.290 [1.685, 2.809]	0.957	0.3385	0.6319
Bupropion vs. Citalopram	1.484 [2.158, 4.749]	1.007	0.314	0.6957	1.885 [1.998, 7.099]	1.8	0.0724	0.2534	2.030 [1.996, 8.224]	2.009	0.0445	0.1775
Bupropion vs. Duloxetine	0.796 [2.020, 1.280]	-0.635	0.5252	0.817	0.684 [2.100, 0.983	-1	0.3165	0.62	0.730 [2.115, 1.125]	-0.825	0.4091	0.6738
Bupropion vs. Mirtazapine	0.731 [2.162, 1.155]	-0.797	0.4255	0.7504	0.505 [2.036, 0.518]	-1.89	0.0592	0.2388	0.495 [1.976, 0.484]	-2.024	0.043	0.1775
Bupropion vs. Trazodone	0.997 [1.704, 1.694]	-0.01	0.9923	0.9923	1.155 [1.606, 2.143]	0.6	0.5515	0.7353	1.133 [1.627, 2.090]	0.504	0.6143	0.8334
Citalopram vs. Duloxetine	0.626 [1.948, 0.763]	-1.379	0.1679	0.6524	0.411 [2.333, 0.395]	-2.06	0.0399	0.2234	0.258 [2.303, 0.153]	-3.185	0.0014	0.0131
Citalopram vs. Mirtazapine	0.438 [1.976, 0.379]	-2.376	0.0175	0.1895	0.358 [1.972, 0.252]	-2.97	0.003	0.042	0.310 [1.988, 0.191]	-3.341	0.0008	0.0112
Citalopram vs. Trazodone	0.689 [1.756, 0.833]	-1.299	0.1939	0.6524	0.749 [1.721, 0.965]	-1.05	0.2955	0.62	0.752 [1.749, 0.988]	-1.002	0.3162	0.6319
Duloxetine vs. Mirtazapine	0.924 [2.024, 1.728]	-0.22	0.8262	0.9253	1.301 [2.143, 3.622]	0.68	0.4991	0.6987	1.184 [2.217, 3.105]	0.416	0.6777	0.8334
Duloxetine vs. Trazodone	1.176 [1.837, 2.540]	0.521	0.6021	0.8429	1.327 [1.820, 3.203]	0.93	0.3543	0.62	1.406 [1.822, 3.600]	1.115	0.265	0.5994
Mirtazapine vs. Trazodone	1.381 [1.713, 3.271]	1.177	0.2394	0.6524	1.484 [1.684, 3.714]	1.49	0.1374	0.3853	1.513 [1.694, 3.873]	1.538	0.124	0.3279

Numbers in brackets indicate the 99% confidence intervals. In drug pairs, the first drug is denoted as 1 and the second drug as 0. A positive estimate indicates that the first drug in the pair increases significant adverse events (SREs).

OR, Odds ratio; lb, Lower bound; up, Upper bound; Adjusted FDR value, Adjusted false discovery rate value; SDoH, Social determinants of health.

**Figure 4:**
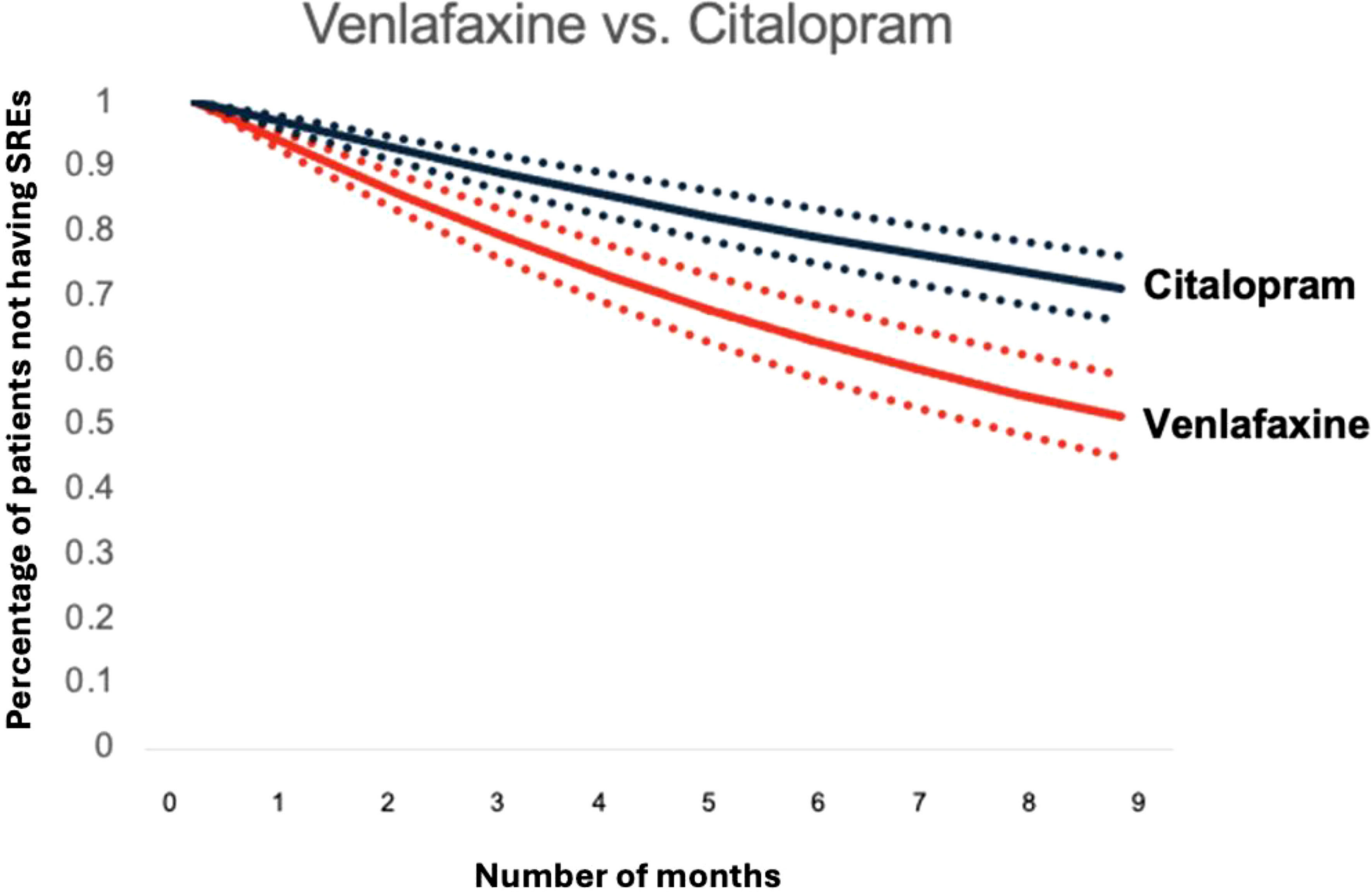
Standardized survival curve based on head-to-head comparisons of venlafaxine and citalopram. The curve shows significant differences in survival rates compared between venlafaxine and citalopram.

**Figure 5:**
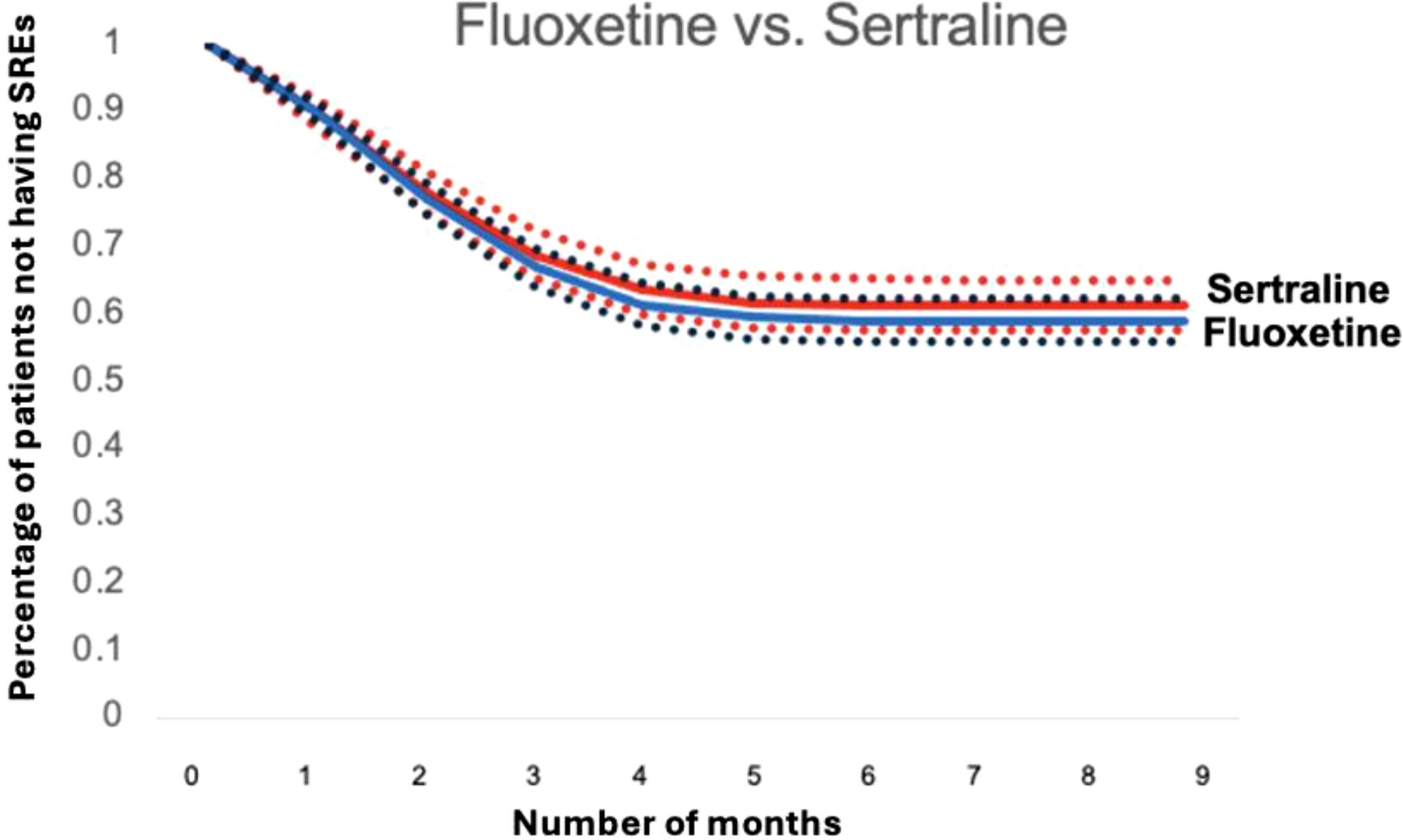
Standardized survival curve based on head-to-head comparisons of fluoxetine and sertraline. The curve shows that both fluoxetine and sertraline have statistically same survival rates when compared.

**Figures 4** and **5** depict standardized survival curves comparing venlafaxine to citalopram (highlighting a statistically significant difference) and fluoxetine to sertraline (indicating a statistically insignificant difference), respectively. These curves illustrate time to target outcome (SREs) adjusted for covariates, including comorbidities, social determinants of health (SDoH), and concurrent medications. The Y-axis, labeled ‘Percentage of patients not having SREs,’ reflects the proportion of patients who have not encountered an SRE at each point in time. It’s important to note that while these survival curves provide adjusted outcomes, they may not precisely reflect the SREs observed in direct head-to-head comparisons. The curves were generated using the methodology outlined by Danaei et al. (32), employing parameters derived from a pooled logistic model to estimate each survival curve (37).

## 4 Discussion

To comprehensively evaluate the impact of antidepressants on the risk of significant adverse events (SREs) in PTSD patients across a diverse population, we utilized electronic medical records from the UPMC health system spanning from January 2009 to October 2020. Leveraging patient-level data, our study aimed to: (1) identify specific antidepressants associated with reduced incidence of SREs among PTSD patients; and (2) adjust for the effects of social determinants of health and other relevant medication use on SRE risk. These analyses significantly expand upon the FDA’s initial assessments, encompassing complete longitudinal records for each patient and providing parallel analyses across diverse populations. Our approach seeks to provide clinicians with a more precise risk-benefit assessment when considering antidepressant use to mitigate SRE risk.

Antidepressants for PTSD treatment have been relatively underexplored, particularly regarding their impact on SREs. Common comorbidities among combat veterans and civilians with PTSD include alcohol use disorder, depression, anxiety disorders, conduct disorder, and substance abuse disorders other than alcohol (42). Chronic use of alcohol, nicotine, and other substances can reduce serotonin levels in the brain (43). Despite the potential benefits of SSRIs in terms of tolerability, treatment adherence, cost-effectiveness, and general safety, their therapeutic advantages for individuals with comorbid mental disorders, suicidal ideation, and addiction remain inconclusive due to limited research in this area. Our study aimed to address this gap, with existing literature suggesting promising efficacy of SSRIs in treating PTSD. Notably, citalopram treatment demonstrated statistically fewer SREs compared to other commonly prescribed antidepressants such as trazodone and sertraline (see **Table 1**). Conversely, bupropion and duloxetine demonstrated statistically higher SRE compared to citalopram, highlighting potential variations in suicide risk among commonly used antidepressants in the PTSD population.

Antidepressants, approved and off-label, play a critical role in managing psychiatric disorders. Research indicates significant reductions in suicide risk measures specifically associated with fluoxetine, though generalization to other antidepressants requires further investigation (44). Another study examined the impact of citalopram microinjections into brain regions implicated in fear response, revealing significant reductions in conditioned fear-induced freezing behavior, thereby supporting serotonin’s role in anxiety reduction through enhanced neurotransmission in PTSD patients (45). Given the diverse indications of antidepressants, patients with PTSD and comorbid disorders such as substance use disorder, alcohol use disorder, anxiety, and depression may benefit from selecting an antidepressant that lowers the risk of SREs. In these scenarios, citalopram may emerge as a preferred option for prescribers considering antidepressant treatment for individuals at high risk for SREs among PTSD patients, despite its FDA approval solely for depression in adults (46). Its off-label use in conditions like alcohol use disorder, coronary arteriosclerosis, obsessive-compulsive disorder, panic disorder, postmenopausal flushing, and premenstrual dysphoric disorder highlights the need for careful consideration of efficacy and safety in the context of PTSD and associated disorders (47). A 2000 study conducted with 14 PTSD patients suggested that citalopram may be effective in alleviating key symptoms of PTSD (48). However, further large-scale studies are necessary to examine citalopram’s impact on multiple PTSD outcomes.

Our study acknowledges several limitations. First, the definition of SREs in our study encompassed suicidal ideation, suicide attempts, and suicide deaths. Due to the low incidence of each type of SRE, the statistical power for analyzing each subcategory was limited. Furthermore, the retrospective nature of our study relied on documentation within medical records, which could have resulted in underreporting of SREs if healthcare providers were unaware of or failed to document an SRE. Second, we did not account for the severity of illness at baseline. As a result, it is not possible to determine if patients treated with certain medications were ill initially. While capturing baseline severity is challenging, we included other mental health-related comorbidities, which may serve as indirect indicators of illness severity. Third, there is no information regarding treatment response in our dataset. Additionally, the complexity of concurrent psychosocial therapies for PTSD management posed practical challenges in controlling for all treatments, and these were not included in the analyses. It is possible that one group received more evidence-based psychotherapy for PTSD or other treatments than another group, potentially influencing the outcomes. Adjustments for comorbidities were based on aggregated logistics derived from baseline information to mitigate confounding variables. Although our study aimed to replicate a randomized controlled trial, the retrospective design precluded controlling for time-dependent confounding variables as effectively as a prospective study. Ethical and feasibility constraints further limited our ability to gather additional information beyond what was available in the medical records. Lastly, the limited sample size of fewer than 100 patients precluded separate analysis for paroxetine, and the statistical power may have been insufficient to detect significant differences between certain treatment comparison groups. However, stringent inclusion criteria were applied to ensure consistency of antidepressant medication exposure during the 60-day “washout” period prior to initiating the target medication.

## Data Availability

The raw data supporting the conclusions of this article will be made available by the authors, without undue reservation.
