# Supplementary material for: Emulating a randomized clinical trial with real-world data to evaluate the effect of antidepressant use in PTSD patients with high suicide risk
Source: Front Psychiatry. 2025 Jan 28;15:1526488. doi: 10.3389/fpsyt.2024.1526488 (PMC11811752; doi:10.3389/fpsyt.2024.1526488)
Supplement: Supplementary file 1 [file DataSheet1.docx]

Supplementary Material

# Appendix A: Diagnosis Codes

PTSD:

309.81, F43.10, F43.11, F43.12

Suicide-related events:

V62.84,R45.851,E950.3,E956,E950.4,E950.0,E958.8,T14.91,E950.9,E958.9,T14.91XA,E950.5,E950.2,E953.0,E958.1,E953.8,E950.1,E950.7,E952.0,E958.0,E957.1,E957.0,E958.5,E952.1,E955.4,T14.91XD,E950.6,E953.9,E955.0,E957.9,E958.7,E958.3,E954,T14.91XS,E951.0,E951.8,E952.8,E953.1,E955.1,E958.6,E958.2,E955.9,E955.2,X83.8XXA,T42.4X2A,T43.592A,T39.1X2A,X78.8XXA,X78.9XXD,T42.6X2A,X78.9XXA,T43.222A,T50.902A,X83.8XXD,T39.312A,T43.212A,T45.0X2A,X78.1XXA,X78.8XXD,T50.992A,T40.2X2A,T43.292A,T43.012A,T39.012A,T42.8X2A,X78.0XXA,T51.0X2A,T40.5X2A,T40.4X2A,T40.1X2A,T44.7X2A,T38.3X2A,T44.6X2A,T48.1X2A,T46.5X2A,T71.162A,T48.3X2A,T43.022A,T44.3X2A,T50.902D,X79.XXXA,T65.92XA,X78.1XXD,T51.92XA,T42.1X2A,T65.892A,T56.892A,T43.622A,X80.XXXA,T42.4X2D,X78.0XXD,T42.72XA,T43.3X2A,X76.XXXD,T48.4X2A,T51.2X2A,T46.4X2A,T39.1X2D,T40.7X2A,T54.92XA,T40.602A,T45.512A,X76.XXXA,T43.222D,T39.392A,T47.1X2A,T50.902S,X74.9XXD,T39.092A,T38.1X2A,X74.9XXA,T39.312D,T38.892A,T43.612A,X82.8XXA,T42.6X2D,T43.632A,T46.1X2A,T45.0X2D,T50.992D,T54.2X2A,T40.3X2A,T39.012D,T43.4X2A,T58.02XA,T43.592D,X81.0XXA,T43.202A,T43.8X2A,T44.992A,T45.2X2A,T40.1X2D,T41.292A,T50.2X2A,T48.6X2A,T50.7X2A,T49.0X2A,T46.3X2A,T42.0X2A,T36.1X2A,T36.0X2A,X74.9XXS,X72.XXXD,T43.012D,T51.8X2A,T51.0X2D,T54.92XS,T54.3X2A,T65.892D,T65.92XD,T65.222D,T50.3X2A,T48.5X2A,T47.0X2A,T46.6X2A,T65.92XS,T54.1X2A,T52.4X2A,T52.0X2A,T55.1X2A,T59.892A,T42.4X2S,T42.3X2A,T36.3X2A,T37.8X2A,T38.2X2A,T38.3X2D,T40.992A,T40.8X2A,T44.4X2A,T43.692A,T45.2X2D,T44.7X2D,T43.502A,T71.192A,X79.XXXD,X83.2XXA,X83.8XXS,X72.XXXA,X71.9XXA,T48.202A,T40.2X2D,X74.8XXS,T48.3X2D,T39.8X2A,T47.4X2A,T47.6X2A,T50.6X2A,T49.6X2D,T43.3X2D,T50.5X2A,X74.01XA,X73.0XXA,T49.6X2A,X72.XXXS,X78.9XXS,T39.92XA,X80.XXXD,X81.8XXA,T39.4X2A,X77.8XXA,T50.2X2D,T43.622D,T43.292D,T45.4X2A,T46.0X2A,T41.3X2A,T42.5X2A,T42.6X2,T46.7X2A,T46.8X2A,T46.5X2D,T43.1X2A,T43.92XA,T40.5X2D,X71.0XXS,X71.3XXA,X71.8XXA,T43.212D,T46.2X2A,T40.8X2D,T40.602D,T43.022D,T44.1X2A,T46.4X2D,T65.222S,T62.0X2A,T71.162D,T51.1X2A,T51.2X2D,T51.2X2S,T52.8X2A,T51.92XD,T50.8X2A,T56.892D,T58.92XA,T54.3X2S,T54.3X2D,T54.0X2A,T55.0X2A,T36.0X2D,T36.4X2A,T38.5X2A,T36.8X2A,T37.5X2A.

# Appendix B: Medications

Table A1: Information on Major concomitant medications at Baseline

| Class of Medications | List of Medications |
| --- | --- |
| Antipsychotics | Aripiprazole, Asenapine, Brexpiprazole, Cariprazine, Chlorpromazine, Clozapine, Fluphenazine, Haloperidol, Iloperidone, Isopropamide, Loxapine, Lurasidone, Mesoridazine, Molindone, Olanzapine, Paliperidone, Perphenazine, Pimavanserin, Promazine, Quetiapine, Risperidone, Thioridazine, Thiothixene, Trifluoperazine, Ziprasidone |
| Benzodiazepines | Alprazolam, Chlordiazepoxide, Clonazepam, Clorazepate, Diazepam, Estazolam, Flurazepam, Lorazepam, Midazolam, Oxazepam, Temazepam, Triazolam |
| Pain Medications | Oxycodone, Fentanyl, Codeine, Hydrocodone, Hydromorphone, Meperidine, Morphine, Tramadol, Gabapentin, Pregabalin |
